# *OCT4* Expression in Gliomas Is Dependent on Cell Metabolism

**DOI:** 10.3390/cimb46020070

**Published:** 2024-01-25

**Authors:** Andrey Volnitskiy, Konstantin Shabalin, Rimma Pantina, Elena Varfolomeeva, Roman Kovalev, Vladimir Burdakov, Svetlana Emelianova, Luiza Garaeva, Alexander Yakimov, Marina Sogoyan, Michael Filatov, Andrey L. Konevega, Tatiana Shtam

**Affiliations:** 1 Petersburg Nuclear Physics Institute Named by B.P. Konstantinov of National Research Centre “Kurchatov Institute”, Orlova Roscha 1, 188300 Gatchina, Russiavarfolomeeva_ey@pnpi.nrcki.ru (E.V.); kovalev_ra@pnpi.nrcki.ru (R.K.); konevega_al@pnpi.nrcki.ru (A.L.K.); 2 Institute of Biomedical Systems and Biotechnologies, Peter the Great St. Petersburg Polytechnic University, Politehnicheskaya 29, 195251 St. Petersburg, Russia; 3 H.Turner National Medical Research Center for Children’s Orthopedics and Trauma Surgery of the Ministry of Health of the Russian Federation, Parkovaya 64-68, Pushkin, 196603 St. Petersburg, Russia; sogoyanmarina@mail.ru; 4 National Research Center “Kurchatov Institute”, Akademika Kurchatova pl. 1, 123182 Moscow, Russia; 5 Institute of Cytology, Russian Academy of Sciences, Tikhoretsky Ave. 4, 194064 St. Petersburg, Russia

**Keywords:** glioma, *OCT4*, α-KG, metabolic cell reprogramming

## Abstract

The OCT4 transcription factor is necessary to maintain cell stemness in the early stages of embryogenesis and is involved in the formation of induced pluripotent stem cells, but its role in oncogenesis is not yet entirely clear. In this work, *OCT4* expression was investigated in malignant gliomas. Twenty glioma cell lines and a sample of normal adult brain tissue were used. *OCT4* expression was found in all studied glioma cell lines but was not detected in normal adult brain tissue. For one of these lines, *OCT4* knockdown caused tumor cell death. By varying the culture conditions of these cells, we unexpectedly found that *OCT4* expression increased when cells were incubated in serum-free medium, and this effect was significantly enhanced in serum-free and L-glutamine-free medium. L-glutamine and the Krebs cycle, which is slowed down in serum-free medium according to our NMR data, are sources of α-KG. Thus, our data indicate that *OCT4* expression in gliomas may be regulated by the α-KG-dependent metabolic reprogramming of cells.

## 1. Introduction

Malignant gliomas are invasive and rapidly progressing primary brain tumors that lead to the quick death of patients. The development of gliomas is associated with a loss of cell differentiation, anaplasia. A population of cancer stem cells capable of forming a tumor and supporting its growth was found in malignant gliomas. These cells expressed stemness markers, could grow as spheres in a serum-free medium with growth factors EGF and FGF-2 [1,2] and formed tumors in the brain of immunodeficient mice, reproducing histological features of human gliomas [3,4].

The stem cell status is maintained by a special set of transcription factors. OCT4, encoded by the *POU5F1* gene, has an essential place among them. It contains conservative Pou and Homeo domains connected by a flexible loop that allows them to bind to large grooves on opposite sides of DNA. OCT4 recognizes the consensus ATGCAAAT sequence, which is often adjacent to the binding sites of other transcription factors, for example, SOX2, which can form complexes with OCT4. OCT4 regulates the expression of such genes as *FGF4*, *UTF1*, *NANOG*, *SOX2* and *POU5F1* itself [5]. In addition, OCT4 has been shown to be a pioneer factor. It binds to DNA at the site of its entry into and exit from the nucleosome and promotes the removal of nucleosomes, opening gene regulatory regions to other transcription factors [6].

In embryogenesis, *OCT4* expression appears at the eight-cell stage, is absent in the trophectoderm, where it is suppressed by CDX2, is present in all cells of the inner mass of the blastocyst and is involved in the formation of epiblasts and primitive endoderm [7]. *OCT4* is part of a combination of genes capable of converting dermal fibroblasts into induced pluripotent stem cells (iPSCs), which can differentiate into cells of all germ layers [8,9]. Interestingly, transfection of *OCT4* alone was sufficient to convert human neural stem cells into iPSCs [10].

Normally, *OCT4* expression is present only in the early stages of development and is absent in the nervous system, including the brain. It has been shown that *OCT4* expression gradually ceases with a concomitant increase in the methylation status of its promoter during the directed differentiation of human embryonic stem cells into neural stem cells. This process occurs through an intermediate stage that contains both embryonic (including OCT4) and neural stem cell markers [11]. At the same time, *OCT4* expression was detected in gliomas [12,13,14,15,16,17,18,19], indicating that this gene is involved in cell malignancy.

In this work, we investigated the expression of *OCT4* and its role in tumor cell survival in our previously obtained glioma cell lines [20]. By changing cell growing conditions, we unexpectedly discovered that *OCT4* expression in gliomas is associated with the metabolic reprogramming of cells.

## 2. Materials and Methods

### 2.1. Glioma Cell Lines and Normal Adult Brain Tissue

Glioma cell lines A-172 and T98G from the Vertebrate cell culture collection of the Institute of Cytology of the Russian Academy of Sciences, primary and secondary cell cultures of 18 glioblastomas (WHO grade IV), previously obtained in the laboratory of cell biology (National Research Center “Kurchatov Institute”—PNPI), as well as a sample of morphologically normal adult brain tissue adjacent to one of the gliomas [20], were used in the present study.

Cells were grown in DMEM/F12 (1:1) with L-glutamine (BioloT, St. Petersburg, Russia) containing 10% fetal bovine blood serum (Cytiva, HyClone). In a number of experiments, recombinant human growth factors EGF, FGF-2 (PanEco, Moscow, Russia) and PDGF BB (ProSpec, Rehovot, Israel) at a concentration of 20 ng/mL were used instead of serum. Serum and L-glutamine deprivation was carried out in DMEM/F-12 medium (1:1) without glutamine (PanEco, Moscow, Russia), whereas L-glutamine was added to 2 mM in the control. Cells were separated from the substrate with Versene solution. The number of cells was assessed using a LUNA-II Automated Cell Counter (Logos Biosystems, Anyang-Si, Republic of Korea) after mixing the cell suspension with trypan blue (1:1).

A comparative analysis of the proliferative activity of cells incubated in a medium with a standard content of serum and L-glutamine, as well as without serum, L-glutamine or both, was performed in real time using the xCELLigence RTCA DP System (ACEA Biosciences, Inc, Santa Clara, CA, USA). Cells were seeded onto a 16-well E-plate at 5 × 10^3^ per well in a medium with serum and L-glutamine, incubated overnight, washed and, after adding the appropriate medium, incubated for 7 days with a continuous recording of electrical resistance every 15 min. All samples within each experiment were in duplicates.

### 2.2. OCT4 Gene Knockdown

Knockdown was performed using siRNA against *OCT4* (Dharmacon ON-TARGETplus SMARTpool human *POU5F1* siRNA, Lafayette, CO, USA) and control siRNA (Dharmacon ON-TARGETplus control siRNA, Lafayette, CO, USA) at a concentration of 60 fmol/μL, which was introduced into cells using lipofectamine (Thermo Scientific DharmaFECT, Waltham, MA, USA) according to the manufacturer’s instructions. Cells were seeded into 24-well plates at 5 × 10^3^ cells per well, incubated with siRNA for 72 h and then separated from the substrate with Trypsin-Versene solution (BioloT, St. Petersburg, Russia) and reseeded into new 24-well plates at 1/10 suspension per well. Cell survival was assessed 6 days after reseeding, when the cells in the control wells almost reached a monolayer. An MTS test with resazurin and subsequent staining of the wells with crystal violet were carried out according to standard protocols. Fluorescence was detected using an EnSpire Multimode Plate Reader (PerkinElmer, Shelton, Connecticut, USA). The stained plates were photographed using the ChemiDoc system (Bio-Rad, Hercules, CA, USA), and the resulting images were processed in ImageJ. Experiments were carried out in triplicate. To test the efficiency of the knockdown, cells were seeded at 5 × 10^5^ cells per 25 cm^2^ flasks and incubated for 48 h with siRNA at the same concentration, and the protein levels were determined via Western blotting.

### 2.3. Western Blotting of OCT4A Protein

Cells were incubated at 4 °C for 30 min with lysis buffer (10 mM Tris-HCl (pH 8.9), 0.1% Triton X-100, 5 mM PMSF, 5 mM MgCl_2_, 5 units/mL DNase I, 20 mM β-mercaptoethanol). The resulting cell lysates were mixed with 4× standard loading buffer (0.25 M Tris (pH 6.8), 8% SDS, 40% glycerol, 20% β-mercaptoethanol, 0.2% bromophenol blue) in a 3:1 ratio and boiled for 5 min. Total protein levels were determined using the Bradford method to load equal amounts of protein onto the electrophoresis lanes. The electrophoretic separation of proteins was carried out in a 10% polyacrylamide gel containing 0.1% SDS, followed by the transfer of proteins to a PVDF membrane (Thermo Scientific, Waltham, MA, USA). To stain the membranes, we used primary mouse monoclonal antibodies to the OCT4A isoform (BD Pharmingen, San Diego, CA, USA) at a dilution of 1:2000 and GAPDH (Merck Millipore, Burlington, MA, USA) at a dilution of 1:500, as well as secondary goat antibodies against mouse immunoglobulins (Thermo Scientific, Waltham, MA, USA) conjugated with horseradish peroxidase at a dilution of 1:20,000. The chemiluminescent detection of electrophoretic zones was performed using the Clarity Western ECL Blotting Substrate kit (Bio-Rad, Hercules, CA, USA).

### 2.4. RNA Extraction and Real-Time RT-PCR

Total RNA was isolated from 2 × 10^6^ cells using an Aurum total RNA minikit (Bio-Rad, Hercules, CA, USA) and GeneJET RNA Purification Kit (Thermo Scientific, Waltham, MA, USA) according to the manufacturers’ protocols. DNA was removed using DNase-I (Thermo Scientific, Waltham, MA, USA) or 12 M LiCl. The amount of isolated RNA was assessed using a NanoDrop One spectrophotometer (Thermo Scientific, Waltham, MA, USA). Reverse transcription was performed using MMLV reverse transcriptase and primer oligo(dT)_15_ (Bio-Rad iScript cDNA Synthesis Kit, Hercules, CA, USA and Evrogen, Moscow, Russia) according to the manufacturers’ protocols.

The PCR reaction mixture (50 μL) contained 200 μM of each dNTP, 2.5 units of HS-Taq DNA polymerase (Evrogen, Moscow, Russia), 300 nM of each oligonucleotide (Syntol, Moscow, Russia) and 2 μg of cDNA. Sequences of primers and fluorescently labeled probes are shown in Table 1.

The reaction was carried out on DT-322 (DNA-Technology, Moscow, Russia) and CFX96 C1000 (Bio-Rad, Hercules, CA, USA) detecting thermal cyclers for 45 cycles. The program included preheating (1 min at 95 °C), DNA denaturation (15 s at 95 °C), primer annealing and amplification (1 min at 60 °C). The mRNA levels of tested genes were normalized on that of *GAPDH* or *actin β*. The absence of genomic DNA was monitored using RNA samples without reverse transcription.

### 2.5. Confocal Scanning Microscopy and Flow Cytometry

Cells grown on coverslips in Petri dishes or transferred into suspension (10^6^ cells) were fixed with 4% formaldehyde (10 min at 4 °C), washed 2 times with PBS, permeabilized with 0.5% Triton-X100 (10 min at 20 °C), blocked with 3% BSA (15 min at 20 °C), incubated with primary monoclonal mouse antibodies to the OCT4A isoform labeled with Alexa647 (BD Pharmingen, San Diego, CA, USA), diluted with 1% BSA 1:20 (1 h at 20 °C) and washed 3 times with PBS. Negative control samples were supplemented with 1% BSA instead of antibodies. Samples for confocal microscopy were mounted in a solution of DAPI and VECTA SHIELD, and samples for flow cytometry were diluted in 300 μL PBS. Analysis was performed on a Leica TCS SP5X confocal scanning microscope at 60× magnification (Mannheim, Germany) and a CytoFLEX flow cytometer (Beckman Coulter, Brea, CA, USA). All experiments were performed at least three times.

### 2.6. NMR

GCL13 cells were seeded into 25 cm^2^ flasks and grown to a near monolayer. They were then washed to remove serum, and 10 mL DMEM/F-12 (1:1) (BioloT, St. Petersburg, Russia) with or without 10% serum was added. Then, 0.5 mL of the medium was taken from the flasks every day for 7 days and stored at −20 °C. When all samples were collected, they were thawed and purified from protein via incubation with 64% methanol at −20 °C for 30 min and centrifugation at 13,400 rpm for 10 min. Equal volumes of the collected supernatants were dried on a Concentrator plus complete system (Eppendorf, Hamburg, Germany) and dissolved in 600 μL D_2_O containing 1mM imidazole and 0.1 mM TSP as internal standards to determine the chemical shifts, metabolite concentrations and pH.

The spectra were recorded on a DirectDrive 700 NMR spectrometer (Varian, Inc., Palo Alto, CA, USA) containing an HCN probehead with a magnetic field gradient along the Z axis. The PRESAT pulse sequence from the spectrometer pulse program library was used to suppress the signal from the solvent (HDO).

The pulse sequence parameters are as follows: duration of the exciting pulse Pw = 6.7 μs, acquisition time of free induction decay (FID) Taq = 4 s, relaxation delay Rd = 12 s and number of repetitions of the pulse sequence Ns = 16.

The spectra were processed in the MestReNova Version 14.1 program (Mestrelab Research S.L., Santiago, Spain) using automatic phase correction. Baseline correction was performed using cubic splines at specified points in areas where there was no signal. The imidazole signal was used as a reference signal for integration, and the chemical shifts were determined from the position of the TSP signal (δ: 0.0 ppm). The concentrations were determined by integrating the signals δ: 5.25 and 4.66 ppm for glucose, δ: 1.34 ppm for lactate and δ: 2.68 ppm for citrate.

### 2.7. Sequencing of the IDH1 and IDH2 Gene Regions Containing Hot Spots

The primers were selected so that the amplified regions of *IDH1* and *IDH2* contained all the hot spots of these genes. The primer sequences are shown in Table 2.

DNA was isolated from 2 × 10^6^ cells using the KR-012 DNA isolation reagent kit (Omnix, St. Petersburg, Russia) according to the manufacturer’s protocol. The PCR reaction mixture (60 μL) contained 2.5 units of HS-Taq DNA polymerase (Evrogen, Moscow, Russia), 250 μM of each dNTP, 750 nM of forward and reverse primers (Alkor Bio, St. Petersburg, Russia) and 14 ng of DNA. The reaction was carried out on a T100 Thermal Cycler (Bio-Rad, Hercules, CA, USA) for 35 cycles. The program included preheating (5 min at 95 °C), DNA denaturation (30 s at 95 °C), primer annealing (25 s at 60 °C) and amplification (45 s at 72 °C). The size of the resulting fragments was confirmed via electrophoresis in a 6% acrylamide gel followed by staining with ethidium bromide solution.

The sequencing of DNA fragments was carried out using the Sanger method at the Evrogen (Moscow, Russia). The results were analyzed using the SnapGene Viewer 6.0.7 program.

### 2.8. Statistical Data Processing

Statistical data analysis was performed using GraphPad Prism 9.5.1 software. The processing of flow cytometry data and their visualization was carried out using the freely available Floreada.io plugin. The error in measuring the relative levels of *OCT4A* and *OCT4B* RNA for gliomas and norm was calculated through the relative error of the 2^dCt^ function and the mean absolute error for the difference in threshold cycles of the reference and target genes, ln2*MAE(dCt). Error bars in other histograms indicate the standard deviation for at least three independent experiments. Data in histograms were compared using the nonparametric Mann–Whitney U test or a one-way ANOVA followed by a Tukey’s HSD test. Values of *p* < 0.05 were accepted as statistically significant differences.

## 3. Results

### 3.1. Expression of OCT4 Is Observed in Malignant Gliomas but Is Absent in Norm

**Figure 1:**
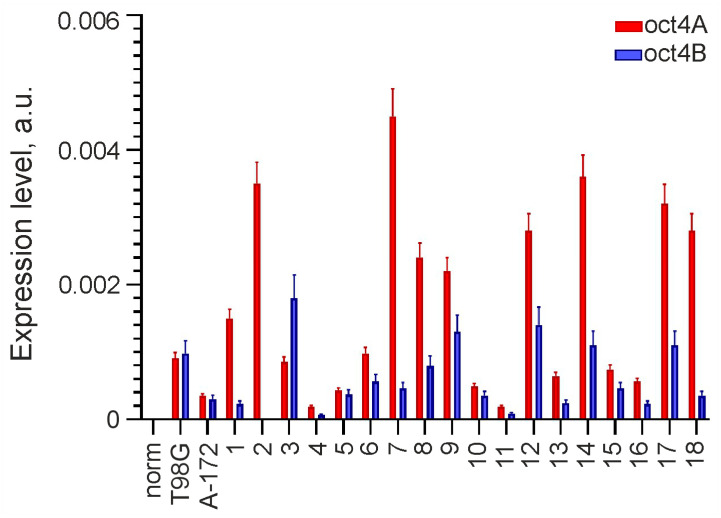
Expression of OCT4 in glioma cell lines and normal adult brain tissue. OCT4 mRNA is represented by isoforms OCT4A and OCT4B, and its levels are normalized to that of GAPDH. GCL2 and GCL13 correspond to numbers 2 and 13 on the histogram.

Analysis of *OCT4* expression was performed using real-time RT-PCR on 20 glioma lines, including A-172, T98G and primary cultures, and one sample of normal adult brain tissue adjacent to one of the gliomas. *OCT4* expression was found in all studied gliomas but was not detected in norm. In 19 of 20 gliomas, *OCT4* RNA was represented by both the *OCT4A* isoform encoding the transcription factor and the *OCT4B* isoform encoding the protein unable to bind DNA. The *OCT4A* isoform alone was detected in only one line (GCL2). The results are presented as a histogram in Figure 1.

### 3.2. OCT4 Knockdown can Lead to Glioma Cell Death

**Figure 2:**
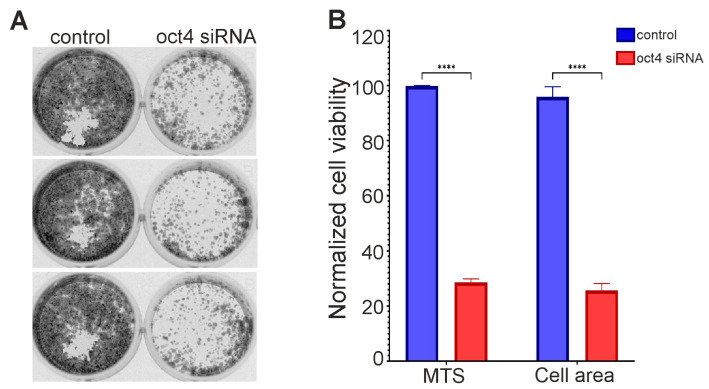
Knockdown of OCT4 in GCL13 cells: (**A**) images of wells with cells stained with crystal violet after incubation with siRNA for 72 h and reseeding; (**B**) cell survival assessed by MTS fluorescence or cell layer area stained with crystal violet. The significance of differences between groups was determined using the Mann–Whitney U test: **** *p* < 0.00005. All experiments were performed at least three times.

Several primary cultures produced well-growing secondary lines. In one of them, the knockdown of *OCT4* significantly reduced cell survival relative to the control (Figure 2). This line is designated here as GCL13 according to the order in the histogram of Figure 1. Images of wells with cells stained with crystal violet after incubation with siRNA for 72 h and reseeding are shown in Figure 2A. After *OCT4* knockdown, cell survival, as measured by MTS fluorescence or the crystal violet-stained cell layer area, was reduced by more than 3-fold relative to the control (Figure 2B). A Western blot showed decreased OCT4A protein levels in GCL13 cells after incubation with siRNA for 48 h (data not presented here). The GCL13 was chosen as a model for further study of the function of OCT4 in gliomas.

### 3.3. Expression of OCT4 in Gliomas Depends on Cell Culture Conditions

The growth factors EGF and FGF-2 are used to grow neural stem cells in culture instead of serum, which induces cell differentiation [21,22]. The growth of glioblastomas of the proneuronal subtype depends on PDGFRA, so another growth factor, PDGF, is used for their cultivation [23]. It has been shown that under these conditions, malignant glioma cells can restore *OCT4* expression [23,24].

**Figure 3:**
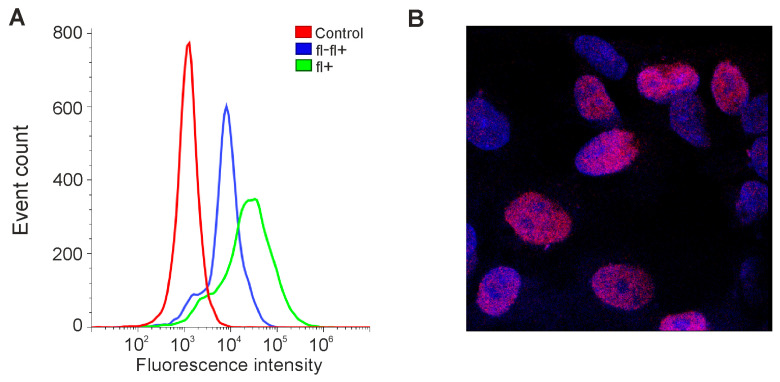
Detection of OCT4A protein in PA-1 cells. (**A**) Flow cytometry: control—here and further, incubation without antibodies; fl-fl+—incubation first with unlabeled and then with fluorescently labeled antibodies to OCT4; fl+—incubation only with fluorescently labeled antibodies to OCT4. (**B**) Confocal microscopy: here and further, blue—DAPI; red—OCT4A.

We decided to test whether the expression level of *OCT4* could be increased in our lines in this way. GCL13 cells were incubated for 48 h in serum-free DMEM/F12 supplemented with EGF and FGF-2 or PDGF BB alone at a concentration of 20 ng/mL for each factor. Cells incubated in medium with 10% serum or without serum were used as a control. Cells were then stained with a primary mouse monoclonal antibody to OCT4A labeled Alexa647, and OCT4A levels were detected via flow cytometry and confocal microscopy. Human teratocarcinoma line PA-1 was used as a positive control for antibody specificity (Figure 3).

**Figure 4:**
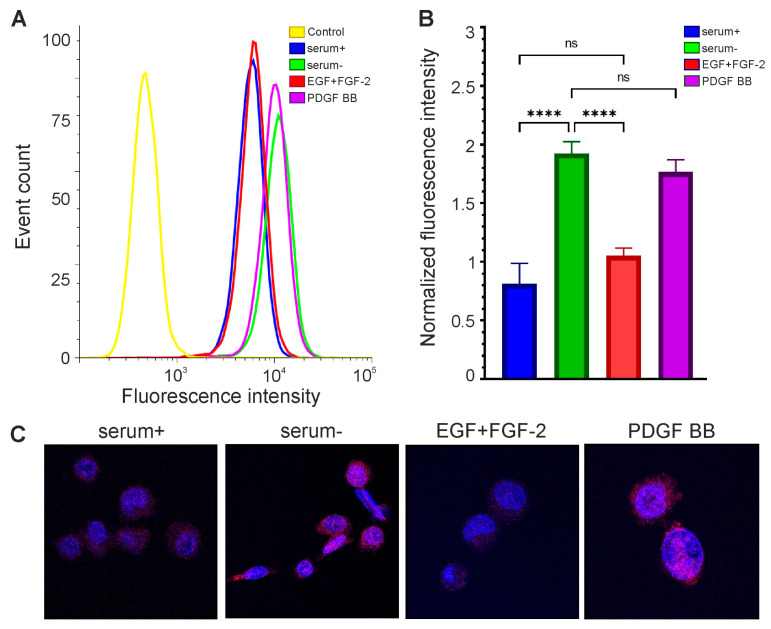
The level of OCT4A protein in glioma cells depends on their growth conditions. Flow cytometry (**A**,**B**) and confocal microscopy (**C**) are performed on GCL13 cells incubated with 10% serum (serum+), no serum (serum-), growth factors EGF and FGF-2 (EGF+FGF-2), or PDGF BB alone (PDGF BB) for 48 h. Panel (**B**) shows the average fluorescence intensity. The significance of differences between groups was determined via a one-way ANOVA followed by a Tukey’s HSD test: **** *p* < 0.00005, ns—nonsignificant (*p* > 0.05). All experiments were performed at least three times.

As expected, in the PA-1 line, OCT4A protein was localized in the nuclei, and its levels varied in different cells (Figure 3B). If cells were incubated first with unlabeled and then with labeled antibodies against OCT4A, the level of fluorescence dropped, confirming the specificity of the selected antibodies (Figure 3A). At the same time, in glioma GCL13, stained with the same antibodies, OCT4A could be located in the nuclei but was mainly detected in the cytoplasm evenly in all cells (Figure 4C). Examples of the detection of OCT4A protein in some other glioma cell lines (GCL12, GCL15, GCL16) via confocal microscopy are shown in Supplementary Figure S1.

The addition of growth factors EGF and FGF-2 did not increase OCT4A levels in GCL13 cells relative to the 10% serum control (Figure 4). At the same time, the level of OCT4A was significantly increased in serum-free medium (Figure 4). In serum-free medium supplemented with PDGF BB, OCT4A levels were almost the same as in serum-free medium, indicating that this growth factor did not affect *OCT4* expression (Figure 4). The localization of OCT4A did not change in any case (Figure 4C). Thus, incubation in serum-free medium was sufficient to increase *OCT4* expression in this culture, while the growth factors EGF and FGF-2 reduced it to the basal level.

### 3.4. In Serum-Free Medium, Glucose Is Absorbed More Slowly, and the Levels of Intermediates Involved in Glycolysis and the Krebs Cycle Are Reduced

Some metabolites are coenzymes for proteins that regulate gene expression and participate in the metabolic reprogramming of cells. For example, α-ketoglutarate (α-KG) supports the activity of 5′-methylcytosine hydroxylases of the ten-eleven translocation (TET) family and histone demethylases with the JmjC domain, which change the state of chromatin through the demethylation of DNA and histones [25,26]. We noticed that in the absence of serum, cells oxidized the medium with lactic acid much more slowly than in a medium with 10% serum or the growth factors EGF and FGF2. Thus, the increase in oct4 expression that we observed could be due to the metabolic reprogramming of the cells.

To investigate how transfer to a serum-free medium changes the metabolic state of the cells, condensed medium from GCL13 cells incubated with or without 10% serum was collected every day for a week and then analyzed via NMR. Peaks corresponding to glucose, lactate (a marker of glycolysis) and citrate (a marker of the Krebs cycle) were well resolved in the resulting spectra, so we focused on these metabolites.

**Figure 5:**
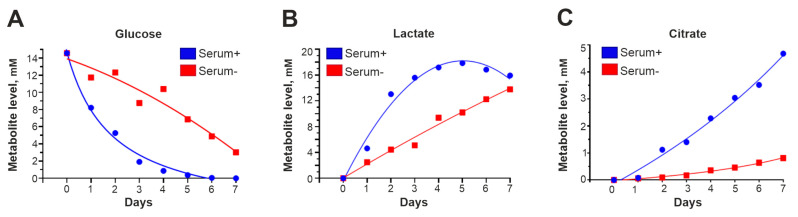
Cell metabolism is slowed in the absence of serum. The graphs in panels (**A**–**C**) show how the levels of glucose, lactic acid and citric acid in condensed medium with 10% serum (serum+) or without serum (serum-) changed during the week.

According to the NMR data, in condensed medium without serum, glucose levels decreased (Figure 5A), and levels of lactate (Figure 5B) and citrate (Figure 5C) accumulated significantly more slowly than in medium with 10% serum. Serum-free medium lacks insulin and other growth factors that stimulate glucose uptake and energy metabolism, including glycolysis and the Krebs cycle. Thus, the rate of these processes is reduced, so the levels of associated intermediates, including α-KG, are also reduced.

### 3.5. GCL13 Does Not Have Mutations in the Hot Spots of the IDH1 and IDH2 Genes

Isocitrate dehydrogenases IDH1 and IDH2 normally catalyze the reversible conversion of isocitrate to α-ketoglutarate (α-KG) in the Krebs cycle. Malignant gliomas may contain mutations in the genes of these enzymes [27]. These mutations are detected in most WHO grade II and III gliomas and secondary glioblastomas derived from them but are almost never found in primary glioblastomas [28]. All identified mutations are located in the fourth exons of these genes and lead to substitutions in one of the three conserved arginine residues in the active site of the enzymes: R100, R109 and R132 in IDH1 and R140, R149 and R172 in IDH2 [28]. Such enzymes lose the ability to bind isocitrate and can only carry out the incorrect reverse reaction, converting α-KG to D-2hydroxyglutarate (2HG) but not to isocitrate [29]. This results in the suppression of the activity of α-KG-dependent dioxygenases, due to a decrease in the level of α-KG and the accumulation of 2HG, which competitively inhibits these enzymes [26].

**Figure 6:**
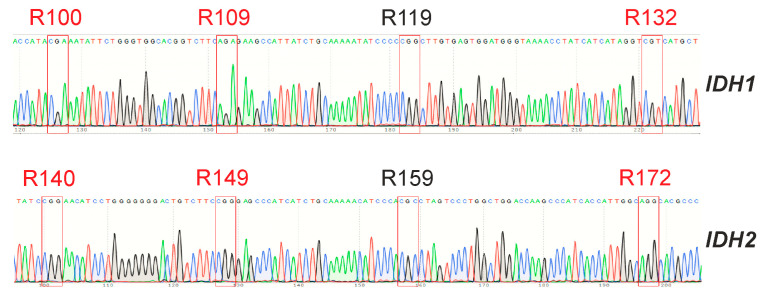
Sequences of regions of the IDH1 and IDH2 genes containing hot spots for GCL13. Hotspot triplets corresponding to conserved arginine residues that can be replaced are outlined in red.

For the GCL13 line, the status of the *IDH1* and *IDH2* genes was determined. Sequencing results are presented in Figure 6. The obtained data did not differ from the reference sequences of the corresponding fragments of these genes from the NCBI database (NG_023319.2, NG_23302.1). Thus, hot spot mutations in these genes were not identified.

### 3.6. Expression of OCT4 Increases in Glioma Cells Incubated in Medium without Serum and L-Glutamine

The sources of α-KG are the Krebs cycle and L-glutamine. If *OCT4* expression is negatively regulated by α-KG, then it should increase in serum-free and L-glutamine-free medium, since both pathways for the synthesis of this metabolite would be suppressed.

**Figure 7:**
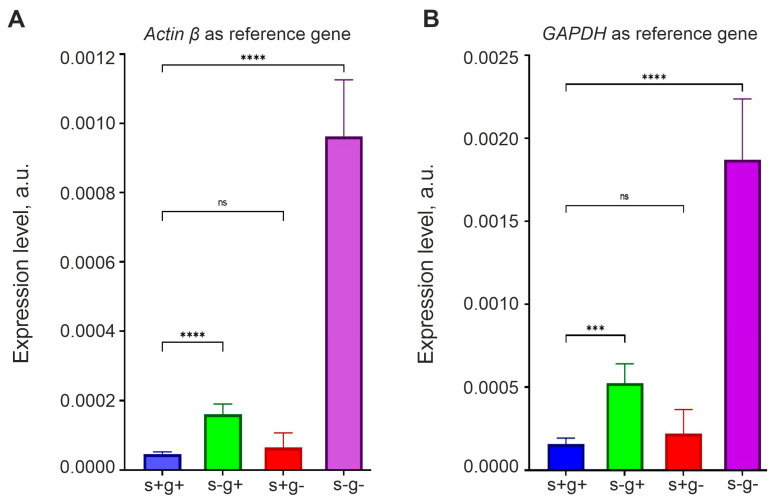
Serum and L-glutamine deprivation enhances OCT4 expression in glioma cells. RT-PCR was performed on GCL13 cells incubated for 48 h in medium with serum and L-glutamine (s+g+), without serum (s-g+) or L-glutamine (s+g-) or both (s-g-). OCT4 mRNA levels are normalized to that of actin β (**A**) or GAPDH (**B**). The significance of differences between groups was determined via a one-way ANOVA followed by a Tukey’s HSD test: ns—*p* > 0.05—no statistical difference, *** *p* < 0.0005, **** *p* < 0.00005. All experiments were performed at least three times.

To test this hypothesis, we incubated GCL13 cells for 48 h in medium that did not contain L-glutamine, serum or both. Cells grown in medium with a standard content of L-glutamine (2 mM) and serum (10%) were used as a control. *OCT4* expression was measured via real-time RT-PCR using primers for the *OCT4A* isoform. We used two reference genes, *actin β* and *GAPGH*, to show that relative *OCT4* mRNA level changes independently of the reference gene to which it is normalized. The results are presented as histograms in Figure 7. The figure shows that serum deprivation (with a slow Krebs cycle!) for 48 h led to a significant increase in *OCT4* mRNA levels, and this effect was enhanced in medium without serum and L-glutamine. In addition, the result did not depend on the choice of the reference gene.

**Figure 8:**
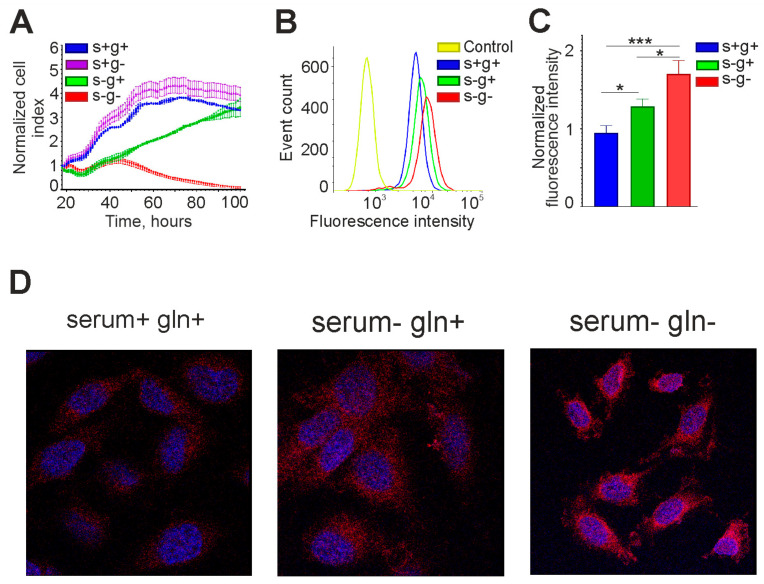
The OCT4A protein level in glioma cells increases upon serum and L-glutamine deprivation. (**A**) Growth curves of GCL13 cells incubated for 3 days in medium with serum and L-glutamine (s+g+), without serum (s-g+), L-glutamine (s+g-), or both (s-g-). The selected fragments of the curves correspond to registration for 3 days. (**B**,**C**) Flow cytometry was performed on GCL13 cells incubated for 48 h in medium with serum (s+g+), without serum (s-g+), or without serum and L-glutamine (s-g-). Panel (**C**) shows the average fluorescence intensity. The significance of differences between groups was determined by one-way ANOVA followed by Tukey’s HSD test: * *p* < 0.05, *** *p* < 0.0005. All experiments were performed at least three times. (**D**) Confocal microscopy was performed on GCL13 cells incubated for 48 h in medium with serum (serum+ gln+), without serum (serum- gln+), or without serum and L-glutamine (serum- gln-).

Interestingly, in medium with 10% serum without L-glutamine, the *OCT4* mRNA level was the same as in the control. L-glutamine is essential for nucleic acid synthesis and cell proliferation, so if it is not available in the environment, it can be synthesized from mitochondrial α-KG, which passes into the cytoplasm [30,31]. In our experiments, cells could not grow long in medium without serum and L-glutamine, whereas in the presence of serum their growth rate was almost the same as in the control (Figure 8A). Thus, L-glutamine was either contained in serum or synthesized from α-KG produced in the Krebs cycle. It is also interesting to note that in the serum-free medium with L-glutamine, cell growth slowed down relative to the control, but did not stop, indicating autocrine regulation (Figure 8A).

The effect of lack of serum and glutamine in the culture medium on OCT4A protein level was also tested. Flow cytometry and confocal microscopy data are presented in Figure 8. OCT4A protein level was increased in serum-free medium, and this effect was further enhanced in serum-free and L-glutamine-free medium, confirming the real-time RT-PCR data.

## 4. Discussion

*OCT4* expression was detected in almost all malignant gliomas of adult patients [13,14,18,19] and 12.5% of pediatric diffuse intrinsic pontine gliomas (DIPGs) [16] but was not found in normal brain tissue [13,19]. The fraction of cells containing OCT4 and its expression level were significantly higher in high-grade gliomas than in low-grade gliomas [13,14,15,17,18,19]. The OCT4 protein was localized predominantly in the nucleus [13,17] but could also be present in the cytoplasm of cells [14,19] and was often detected together with other stem cell markers, for example, SOX2 [14,15,18]. In glioblastomas, an increase in OCT4 protein levels was observed in blood vessels but not in necrotic areas and pseudopalisade cells [19]. In WHO grade III astrocytomas with the R132H mutation in IDH1, *OCT4* expression levels were on average higher than in wild-type gliomas and were associated with a worse patient prognosis [19]. OCT4 has been shown to enhance *SOX2* expression by forming a complex with SOX4 at its enhancer [32]. Knockdown of *OCT4* reduced cell proliferation and their ability to form spheres or tumors in the brains of immunodeficient mice increased sensitivity to temozolomide but did not induce apoptosis [32]. Exogenous expression of *OCT4* in glioblastoma cells significantly enhanced their motility and invasion. At the same time, there was a change in the expression profile of integrins and an increase in focal cell adhesion, as well as the expression and activity of the matrix metalloprotease MMP-13 [33].

In accordance with previously obtained data, we found *OCT4* expression in all studied glioma lines but not in normal adult brain tissue. In almost all lines, *OCT4* RNA was represented by both the *OCT4A* isoform, which encodes a transcription factor, and the *OCT4B* isoform, which is not associated with transcription regulation. At the same time, in contrast to the data of other researchers, the OCT4A protein was mainly present in the cytoplasm and significantly less in the nuclei of glioma cells. The antibodies and staining procedure were tested using the teratocarcinoma model line PA-1, where this protein was detected exclusively in the nuclei. It is possible that gliomas have a mechanism that keeps OCT4A outside the nucleus and thereby limits its activity as a transcription factor. Our discrepancies with previous data may be due to different cell growth conditions. Most studies used paraffin sections of tumors [14,19] or primary cultures initially growing as spheres in serum-free medium supplemented with growth factors EGF and FGF-2 [23,32], whereas our primary cultures grew as a monolayer in medium containing 10% serum. Interestingly, *OCT4* knockdown carried out under these conditions led to cell death in one of our lines, and this result is new for gliomas. Thus, this protein is functionally significant in gliomas and may be required for tumor cell survival. The GCL13 line (also called as Gl-Tr [34]) we obtained will serve as a model for further studying the function of OCT4 in gliomas.

Previous data indicate that *OCT4* expression and its transcriptional activity are associated with the tumor-initiating cell population. For example, *OCT4* expression was detected in tumor cells circulating in the blood of patients with glioblastomas, which could repopulate the site of the original tumor after its surgical removal or give rise to new, more aggressive lesions [35]. In another study, U87 MG cells were modified with a construct containing a promoter with a binding motif for the OCT4 and SOX2 complex, a fluorescent reporter and a gene for an enzyme that activates the replication inhibitor ganciclovir and inoculated into the brains of immunodeficient mice. Treatment with ganciclovir significantly increased the survival of mice but could not completely eliminate fluorescence. If treatment was interrupted, tumor growth continued after a certain time, indicating that some tumor-initiating cells remained dormant [36]. However, according to our observations, OCT4A was present in GCL13 cells grown with 10% serum. In contrast to PA-1, all GCL13 cells in the fields selected on the confocal microscope were uniformly stained for OCT4A. Therefore, in gliomas, *OCT4* expression alone cannot be a specific marker of tumor-initiating cells.

Serum is often used to culture cells, but even in low concentrations it causes their differentiation. To preserve cell stemness, it is replaced with specially selected combinations of growth factors. In a medium containing the growth factors EGF and FGF-2 instead of serum, glioma cells show increased expression of stemness phenotype markers and can form spheres, just like normal neural stem cells [1]. OCT4 protein levels increased in some glioma lines when cells were transferred from 10% serum to these conditions [24]. In another study, freshly resected glioblastoma multiforme cells were grown in a special serum-free medium supplemented with EGF and FGF-2 or PDGFA alone. Expression of *OCT4* and other stem cell markers appeared only after 48 h. Cells lost *OCT4* and *NANOG* expression and the ability to form spheres when the activity of these growth factor receptors, ERK or the transcription factor EGR1 was inhibited [23]. However, according to our data, incubation in serum-free medium itself increased the expression of *OCT4*, while the growth factors EGF and FGF-2 reduced it to the basal level. This can be explained by a decrease in PI3K/Akt/mTOR activity due to the lack of insulin and other growth factors that stimulate this signaling cascade. It has been shown that in gliomas *OCT4* expression is maintained by the transcription factors FOXO1 and FOXO3 and suppressed by PI3K/Akt/mTOR, and its level is significantly enhanced when PI3K is inhibited simultaneously with mTOR or MEK [37]. Interestingly, EGR1 directly activates the expression of the phosphatase PTEN, which inhibits PI3K/Akt/mTOR [38], so the discrepancy between our data and previous work [23] may be resolved by studying the dynamics of the described mechanisms.

In addition, reduced PI3K/Akt/mTOR activity will lead to a slower cell metabolism, including glycolysis and the Krebs cycle. Accordingly, the levels of intermediates involved in these processes should decrease, which we observed for lactic and citric acids using NMR. One of these intermediates is α-KG, which supports the activity of a number of enzymes that regulate gene expression and take part in the metabolic reprogramming of cells. We suggested that a decrease in α-KG levels would cause an increase in *OCT4* expression through epigenetic changes. Since the source of this metabolite is not only the Krebs cycle but also L-glutamine, the simultaneous suppression of both pathways of α-KG synthesis should have significantly enhanced the effect. Using medium without serum and L-glutamine, we indeed observed a significant increase in *OCT4* expression in both RNA and protein, consistent with our hypothesis. Thus, α-KG-dependent cell reprogramming may regulate *OCT4* expression in gliomas, but the enzymes and epigenetic modifications that are involved in this process remain to be elucidated.

## 5. Conclusions

Taken together, our data confirm that *OCT4* expression is present in malignant gliomas but is absent in normal adult brain tissue. In addition, we show for the first time that OCT4 may be required for glioma cell survival. The increase in *OCT4* expression in serum-free and L-glutamine-free medium indicates that this process is regulated by metabolic reprogramming systems that remain to be identified.

## Figures and Tables

**Table 1:** Primers and fluorescently labeled probes.

	Forward Primer	Reverse Primer	Probe (FAM-BHQ1)
Analysis of oct4 expression in gliomas and norm			
*OCT4A*	aagctggagaaggagaagct	cagatggtcgtttggctgaa	actcgagcaatttgccaagctc
*OCT4B*	gttcttacaagtcttctgcct	cagatggtcgtttggctgaa	actcgagcaatttgccaagctc
*GAPDH*	catgggtgtgaaccatgagaa	ggtcatgagtccttccacgat	aacagcctcaagatcatcagcaatgcct
Serum and L-glutamine deprivation			
*OCT4A*	aagctggagaaggagaagct	cagatggtcgtttggctgaa	actcgagcaatttgccaagctcc
*actin β*	atgcagaaggagatcactgc	atactcctgcttgctgatcc	atcattgctcctcctgagcgcaa
*GAPDH*	tcatgggtgtgaaccatgag	atggcatggactgtggtcat	gatcatcagcaatgcctcctgca

**Table 2:** Sequencing primers.

	Forward Primer	Reverse Primer	Fragment Length
*IDH1*	gagaatcgtgatgccaccaa	ttggaaatttctgggccatga	340 bp
*IDH2*	tctggctgtgttgttgcttg	agagacaagaggatggctag	325 bp

## Data Availability

All data generated or analyzed during this study are included in this published article and its Supplementary Information Files.

## Supplementary Materials

The following supporting information can be downloaded at: https://www.mdpi.com/article/10.3390/cimb46020070/s1. Figure S1. Detection of Oct4A protein in glioma cells by confocal microscopy. The cells of some glioma cell lines (GCL13, GCL16, GCL12, GCL15) were immunostained with antibodies for oct4A (red) and stained with DAPY (blue). Primary monoclonal mouse antibodies to oct4A (BD Pharmingen) and then secondary goat antibodies to mouse antibodies labeled with Alexa594 (Invitrogen, Waltham, MA, USA) were used.
