# Supplementary material for: OCT4 Expression in Gliomas Is Dependent on Cell Metabolism
Source: Curr Issues Mol Biol. 2024 Jan 25;46(2):1107–20. doi: 10.3390/cimb46020070 (PMC10887564; doi:10.3390/cimb46020070)
Supplement: Supplementary file 1 [file cimb-46-00070-s001.zip › cimb-2771247-supplementary.pdf]

Supplementary Materials

OCT4 expression in gliomas is dependent on cell metabolism

Andrey Volnitskiy [1,*], Konstantin Shabalin [1], Rimma Pantina [1], Elena Varfolomeeva [1], Roman Kovalev[1], Vladimir Burdakov [1], Svetlana Emelianova [1], Luiza Garaeva [1], Alexander Yakimov [1,2], Marina Sogoyan [3], Michael Filatov [1], Andrey L. Konevega [1,2,4] and Tatiana Shtam [1,4,5,*]

[1] Petersburg Nuclear Physics Institute Named by B.P. Konstantinov of National Research Centre "Kurchatov Institute", Orlova Roscha 1, 188300 Gatchina, Russia; varfolomeeva_ey@pnpi.nrcki.ru (E.V.); kovalev_ra@pnpi.nrcki.ru (R.K.); konevega_al@pnpi.nrcki.ru (A.L.K.)

[2] Institute of Biomedical Systems and Biotechnologies, Peter the Great St. Petersburg Polytechnic University, Politehnicheskaya 29, 195251 St. Petersburg, Russia

[3] H.Turner National Medical Research Center for Children's Orthopedics and Trauma Surgery of the Ministry of Health of the Russian Federation, Parkovaya 64-68, Pushkin, 196603 St. Petersburg, Russia; sogoyanmarina@mail.ru

[4] National Research Center "Kurchatov Institute", Akademika Kurchatova pl. 1, 123182 Moscow, Russia

[5] Institute of Cytology, Russian Academy of Sciences, Tikhoretsky Ave. 4, 194064 St. Petersburg, Russia

* Correspondence: volnitskiy_av@pnpi.nrcki.ru or voln.a@yandex.ru (A.V.); shtam_ta@pnpi.nrcki.ru (T.S.)

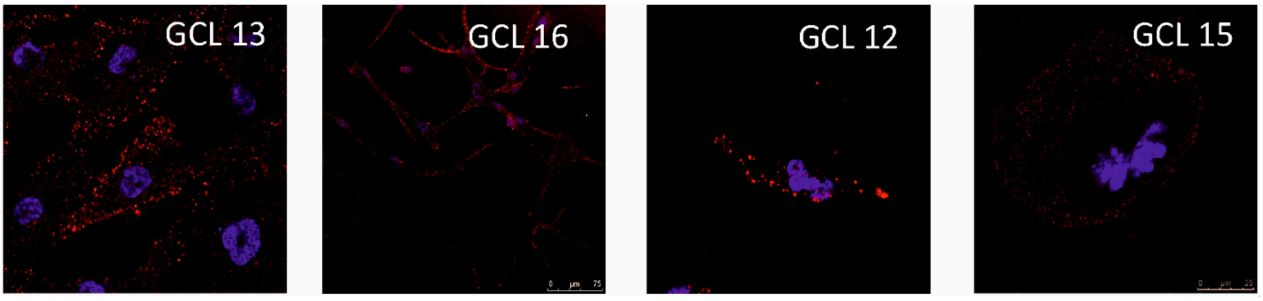

Supplementary Figure S1. ***Detection of Oct4A protein in glioma cells by confocal microscopy.*** *The cells of some glioma cell lines (GCL13, GCL16, GCL12, GCL15) were immunostained with antibodies for oct4A (red) and stained with DAPY (blue). Primary monoclonal mouse antibodies to oct4A (BD Pharmingen) and then secondary goat antibodies to mouse antibodies labeled with Alexa594 (Invitrogen) were used.*
